# Temporal bone histopathology – idiopathic sudden hearing loss

**DOI:** 10.1016/S1808-8694(15)30770-9

**Published:** 2015-10-19

**Authors:** Fayez Bahmad

**Affiliations:** 1 MD. PhD student – FM-UnB, Researcher at the Department of Otology – Massachusetts Eye & Ear Infirmary – Harvard Medical School, Boston, MA, EUA Department of Otolaryngology – University Hospital – Brasília, Brasília, Distrito Federal, Brasil 2 Otology Department, Massachussetts Eye & Ear Infirmary, Boston, Massachusetts, EUA. 243 Charles St., Massachusetts Eye & Ear Infirmary, 4 th Floor, Room 468, ZC 02114, Boston, MA, USA.

**Keywords:** hearing loss, histopathology, sudden, idiopathic

## INTRODUCTION

Idiopathic Sudden Hearing Loss (ISHL) is characterized by a sudden, bilateral or unilateral hearing deficit, which develops within a period of 72 hours and still poses a diagnostic and treatment challenge to otolaryngologists. Its cause and pathogenesis are still unknown. Among the theories proposed to explain its pathogenesis, we list cochlear viral infection, vascular occlusion and membrane rupture^1^^,^^2^.

## CASE PRESENTATION

This patient had left side idiopathic sudden hearing loss at 40 years of age. He reports that when he woke up in the morning, he noticed a high frequency tinnitus on his left ear, he felt irritable and unable to concentrate enough to read the newspaper, as he had done every morning. Some hours later he felt ear fullness, diplacusis and sudden profound hearing loss, and he also had trouble keeping his balance. He did not report upper airway infection symptoms that day, nor the week before. Tonal and vocal audiometry showed profound hearing loss on the left side. His electrocochleogram (EcoG) showed no cochlear potentials on the left side and the electronystagmography (ENG) showed a vestibular function deficit to the right side. He was treated as having sudden sensorineural hearing loss of viral origin, and treatment details are unknown. Tonal and vocal audiometry carried out six months afterwards revealed 20 dB thresholds for frequencies 125, 250 Hz and of 80–90 dB in the high frequencies, with speech recognition of 40%. This patient died at 62 years of age, of cardiac cause. His temporal bones were removed 6 hours after his death and immediately fixed in 10% formalin solution.

## DISCUSSION

### Histopathology report

**Figure 1A:**
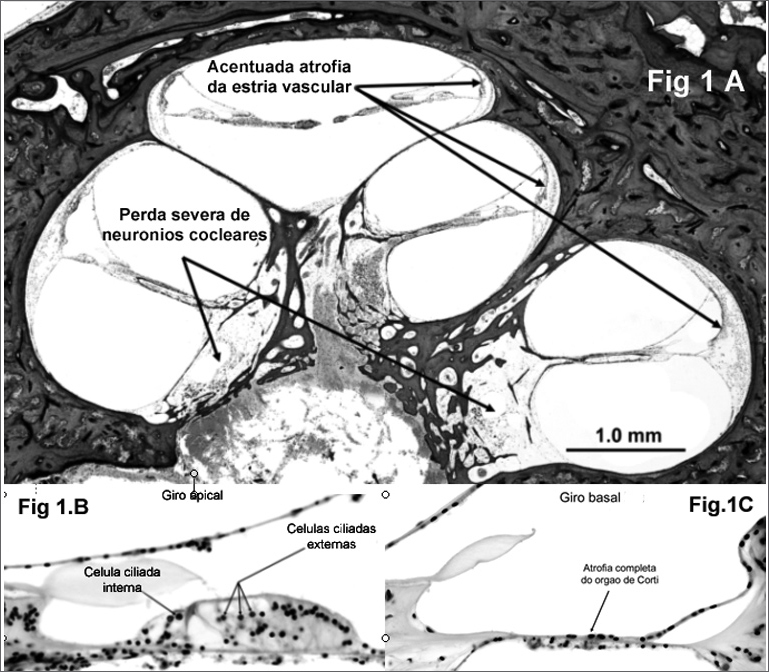
Cochlea overview, showing a reduction in cochlear neurons, especially in the basal turn and marked stria vascularis atrophy in all cochlear turns. **Figure 1B.** Apical turn showing a normal Organ of Corti. Inner and Outer hair cells present. **Figure 1C.** Basal turn with complete Organ of Corti atrophy.

Histopathology analysis showed a very atrophic stria vascularis in all of its turns and mass loss of cochlear neurons in the basal turns, with greater dendritic loss when compared to central axons (neuronal retrograde degeneration), consequent to a loss in the Organ of Corti and hair cells (Figure 1A). Cochlear neurons are abundant (normal for his age) in Rosenthal's canal, which innervates the Organ of Corti in the middle and upper turns, and the cochlear nerve in the internal auditory meatus did not show alterations. The Organ of Corti was absent in most of the basal turn (Figure 1C), while it was intact in the apical turn and the external and internal hair cells were present and can be seen in Figure 1B. Such finding explains the patient's high frequency hearing loss and normal low frequency hearing. (According to the frequencies scale which is based on the cochlear anatomy proposed by Schuknecht in 1993, cochlear lesions in frequencies above 2000 Hz are anatomical located in the basal cochlear turns)^1^. There are no signs of bone neoformation anywhere in the inner ear, nor suggestive signs of cochlear damage by vascular occlusion, and the arterioles and venules responsible for blood flow seemed intact.

## FINAL COMMENTS

This patient developed profound left side sensorineural hearing loss, with a downsloping audiometric pattern which can be explained based on the loss of the Organ of Corti and hair cells in the basal turns. There was no microscopic evidence of viral involvement, vascular occlusion, perilymphatic fistula or any other known cause.
